# Factors associated with contraceptive use among sexually active Nepalese youths in the Kathmandu Valley

**DOI:** 10.1186/s40834-017-0040-y

**Published:** 2017-03-14

**Authors:** Laxmi Tamang, Camille Raynes-Greenow, Kevin McGeechan, Kirsten Black

**Affiliations:** 1 APS Prasuti Tatha Prajanan Swasthya Kendra, Link Marg 1045, Kuleshwor, Kathmandu, Nepal; 2 School of Public Health, Faculty of Medicine, The University of Sydney, Camperdown, NSW 2006 Australia; 3 Discipline of Obstetrics, Gynaecology and Neonatology, Faculty of Medicine, The University of Sydney, Camperdown, NSW 2006 Australia

**Keywords:** Contraceptive, Nepal, Young people

## Abstract

**Background:**

In Nepal, evidence is sparse regarding the use of contraception at first and current relationships among sexually active young people. This study examined the factors associated with modern contraceptive use at first and current sexual relationships.

**Methods:**

A descriptive cross-sectional household survey conducted amongst young Nepalese men and women living in the urban areas of the Kathmandu valley. We used logistic regression to model the relationship between selected independent variables and outcome variables (use at first sexual intercourse and current use of modern contraception) among 492 ever sexually active youth aged 15–24 years.

**Results:**

We found that the key factors associated with current non-use of modern contraceptives among sexually active youth age 15–24 were young age at first sexual contact and a relationship with someone other than a spouse, while significant factor associated with current use of contraception was religion, revealing that Hindu youths having lower odds of use compared to young people who belonged to other religions.

**Conclusions:**

The findings suggest that contraception education should be intensified and directly towards those entering adolescence to encourage youths to adopt contraception at the time of their first sexual relationship. The influence of religion on use of modern contraception needs further exploration.

## Background

Access to safe and effective contraception is essential for optimal sexual and reproductive health [1, 2] and allows for the prevention of unintended pregnancies, improved pregnancy spacing and reduced transmission of sexually transmitted infections and HIV [3–5]. Although overall contraceptive use has increased worldwide, youth access to sexual and reproductive health services and comprehensive sexuality education, that are high-quality, youth-friendly and respect the right to confidentiality, privacy and informed consent remains a challenge in many countries [6–8]. Globally, from a public health perspective there is a widening gap between sexual debut and age of marriage, and increased sexual activity prior to marriage [9], which exposes young people to the risk of unplanned pregnancy [10–12].

The few studies that have been conducted in Nepal on young peoples’ sexual behaviour have found that they experience a wide range of consensual and coercive sexual relationships and that the proportion of sexually experienced unmarried young people is rising [7, 13–17]. Early sexual experimentation, combined with low and irregular use of condoms in Nepalese society places young people at risk of HIV and other sexually transmitted infection [7, 13, 15–17]. Indeed the Nepal Adolescent and Youth Survey 2010/2011 reported that one in six young people aged 15–24 had been involved in risky sexual practices such as oral, anal and paid sex and almost 13% had premarital sex, of which only 46% had used contraception at the time of their first sexual intercourse [15]. The same study revealed that almost one-fifth of young people had their first sexual intercourse unwillingly and forcefully.

As well as being exposed to sexually transmitted infections, unsafe sex exposes young people to the risk of unplanned pregnancy. Across South Asia the uptake of modern contraceptive methods amongst sexually active young people has been poor and early childbearing is common [9, 18]. This is true of women in Nepal where almost one quarter of women give birth by age 18 and nearly half by age 20 [17]. While use of any contraceptive method, including modern contraceptive methods by reproductive aged women married or in a union, is around the average for South Asian countries at 49%, the incidence of unintended pregnancy is higher in Nepalese adolescents (32%) [19]. Furthermore, the 2011 Nepal Demographic Health Survey reported that unmet need for contraception is particularly high among young women aged 15–19 and 20–24, at 41% and 37% respectively compared to women in older age groups 30% or less.

In Nepal, family planning being one of the priority programs of the government modern contraceptive methods both short-acting and long acting reversible contraceptive services are freely available to all reproductive age men and women through public health system. The main aim of the national family planning program is to ensure that individuals and couples are able to fulfil their reproductive needs by using appropriate contraceptive methods based on informed choice.

Following the International Conference on Population and Development held in Cairo in 1994, the government of Nepal committed to promoting and protecting the sexual and reproductive rights of adolescents and youth [20]. Collaborating with national and international agencies the Nepalese government is striving to achieve the development goals that place considerable importance on increasing the use of contraception and reducing the fertility rate, as well as to address gender inequality and women’s empowerment [21]. Understanding the key factors influencing contraceptive use among young sexually active youths who are at risk of unwanted pregnancies is key to the development of effective family planning programs. This study aims to examine the factors associated with contraceptive use at the first sexual intercourse and current relationships among Nepalese youth living in urban areas of the Kathmandu Valley.

## Methods

### Study setting and design

The data were collected from a field survey conducted in the urban areas of the Kathmandu Valley, comprising all five major cities: Kathmandu metropolitan city, Lalitpur sub-metropolitan city, Bhaktapur municipality, Kirtipur municipality, and Thimi municipality. The study was part of a larger study that employed mix-methods, and comprised a household survey, focus group discussions and in-depth interviews aiming to investigate how gendered power relations influence knowledge of SRH and access to, and utilisation of, SRH services among youth in the Kathmandu valley, Nepal. In this paper we present the cross-sectional household survey information. The data were collected using two-stage cluster sampling from the study area. In the first stage of sample selection, primary sampling units (clusters i.e. wards) were selected using a probability proportional-to-size from each study area. We randomly selected a total of 40 clusters from three district urban areas using household and population information from the 2011 Population Census developed by the Central Bureau of Statistics. In the second stage, 35 households in each cluster were selected using a systematic sampling technique. Thus, we needed to visit a total of 1400 households in order to interview the required number of youths.

### Study population

This study was part of the large study among youth thus information was collected from 1400 urban youths, comprising 720 females and 680 males. The sample size for the study was calculated based on the 50% prevalence of young Nepalese women aged 15–24 who had ever had sexual intercourse with 95% confidence intervals, 5% margin of error, 1.6 design effect obtained from those who had had sexual intercourse before age 18 and 10% none response rate [15, 17]. However, for the purpose of this paper only 492 young men and women who had prior sexual experience were included to examine the use of contraceptives at their first and current sexual relationships.

### Questionnaire

The questionnaire consisted of standard items that were adapted from previous studies and validated in similar settings including questions from the Nepal Demographic Health Surveys [15, 22–25]. Some items were modified and added to the questionnaire to suit the context of the study. The questionnaire was finalised after pilot-testing outside the study areas.

Two questionnaires were administered: the household questionnaire and the individual questionnaire. The household questionnaires were used to list all the usual members in the selected households and gathered basic information about the household and characteristics of individuals living there. The individual questionnaire was administered to all youth aged 15–24 by trained female research assistants. The respondents were interviewed face-to-face and provided with an explanation for question items.

### Measures

Respondents were asked if they had ever had engaged in sexual intercourse in their life to ensure they met the “sexually active” requirement for participation in the study. Any participant who answered “no” was not eligible for participation in the study. Lastly, participants were asked if they were currently involved in a relationship where they had regularly have sexual intercourse, and if they answered yes, they were further asked to indicate the length of this relationship.

### Analysis

We applied both univariate and multivariable analyses in this study to examine the association between each of the outcome variables, ‘use of modern contraceptive method at the first sexual contact’ and ‘current use of contraceptive’, and he explanatory variables sex, age, marital status, place of origin, education, ethnicity, religion, occupation, source of income, age at the first sexual intercourse, relationship type, method of participation in the first sexual intercourse, ever discussed about the importance of safe sex with partner, ever had discussed of contraceptive use with partner, and the main source of sexual and reproductive information among sexually active youths who were not planning to have a baby [21, 26–28]. A dichotomous measure indicated whether respondents had used any modern method contraception the first time they had intercourse and whether they were currently using contraceptives. We used the IBM Statistical Package for the Social Sciences (SPSS) software Version 16 (SPSS Inc., Chicago, IL, USA) complex samples procedures to account for the sampling design in the calculation of the association measures. The level of statistical significance was set at a level of *p <* 0.05.

### Ethical approval

Ethical approval was granted by the University of Sydney’s Human Research Ethics Committee and from the Nepal Health Research Council. Informed consent from each study participant was obtained after clear explanation about the purpose of the study. A written informed consent sheet was prepared for the parents or legal guardians of participants less than 16 years to give their signed consent on behalf of their children. The study participants and parents or legal guardians were informed that they had full right to withdraw from the study. Confidentiality of the information was assured by omitting names of study participants from the questionnaire and respondents were interviewed in a separate place to maintain their privacy.

## Results

### Socio-demographic characteristics

Of the total 492 sexually active young men and women, the majority of there were aged 20–24 (77% men and 79% women), had never been married (13% men and 95% women), were urban in origin (56% men and 64% women), and had ever studied at school (98% men and 92% women) women (Table 1). Most of them had the School Leaving Certificate (SLC) or above level of education (84% men and 48% women). More than half were Indo-Aryan by ethnicity (57% men and 56% women) and most were Hindu (79% men and 83% women). Men were significantly more likely than women not to be married (*p* < 0.001), ever studied at school (*p* = 0.011), have obtained a degree in higher education (*p* < 0.001), were students (*p* < 0.001), self-dependent for income (*p* = 0.011), had relationship with girlfriends (*p* < 0.001), participated willingly in a sexual activity (*p* < 0.001), had two or more sexual partners in their life time (*p* < 0.001), ever discussed of contraceptive use (*p* = 0.002) and radio was their major source of SRH information (*p* = 0.002).

**Table 1:** Socio-demographic characteristics of young people aged 15–24 by sex

Background Characteristics	All (n = 492)	Male (n = 247)	Female (n = 245)	*P* value
	No. (%)	No. (%)	No. (%)	
Age (in years):				
15–19	108	57 (23.0)	51 (20.7)	0.605
20–24	384	190 (77.0)	194 (79.3)	
Marital Status:				
Ever married	265	33 (13.4)	233 (95.0)	<0.001*
Never married	226	214 (86.6)	12 (5.0)	
Place of origin:				
Rural	296	139 (56.3)	157 (64.2)	0.187
Urban	196	108 (43.7)	88 (35.8)	
Ever studied at school:				
Yes	469	243 (98.3)	226 (92.2)	0.011*
No	23	4 (1.7)	19 (7.8)	
Education:				
Some secondary and below^a^	168	41 (16.4)	127 (52.0)	<0.001*
SLC and above	324	206 (83.6)	118 (48.0)	
Ethnicity:				
Tibeto-Burmese	214	106 (42.9)	108 (44.3)	0.799
Indo-Aryan	278	141 (57.1)	137 (55.7)	
Religion:				
Hindu	400	197 (79.6)	203 (83.1)	0.408
Non-Hindu^b^	92	50 (20.4)	42 (16.9)	
Occupation:				
Non-students	277	74 (29.7)	203 (83.1)	<0.001*
Students	215	173 (70.3)	42 (16.9)	
Source of income:				
Parents and others	432	205 (83.1)	227 (92.6)	0.012*
Self	60	42 (16.9)	18 (7.4)	
Age at the first sexual intercourse				
12–15	42	20 (8.0)	22 (9.1)	0.051
16–19	306	165 (67.1)	141 (57.5)	
20–24	144	62 (25.0)	82 (33.4)	
Relationship type				
Spouse	255	22 (8.8)	233 (95.0)	
Boy/girlfriend	215	205 (82.9)	10 (4.3)	<0.001*
Familiar person/sex worker^c^	22	20 (8.3)	2 (0.7)	
Type of participation				
Willingly	457	241 (97.7)	216 (88.3)	<0.001*
Unwillingly/forcefully	35	6 (2.3)	29 (11.7)	
No. of sexual partners in life time				
One	451	107 (43.1)	244 (99.7)	<0.001*
Two and more	141	140 (56.9)	1 (0.3)	
Ever discussed of safe sex with partner				
No	120	50 (20.3)	70 (28.4)	0.100
Yes	372	197 (79.7)	175 (71.6)	
Ever discussed of contraceptive use				
No	68	20 (8.2)	48 (19.6)	0.002*
Yes	424	227 (91.8)	197 (80.4)	
Source of SRH information				
Radio	391	212 (85.9)	178 (72.9)	0.002*
Television	70	26 (10.7)	44 (17.9)	
Friends, teachers and others	31	8 (3.4)	23 (9.3)	

^a^No education: 5; Primary 22; ^b^Buddhist: 69; Kirat: 12 Christian: 7; Islam: 4; ^c^Familiar person: 19, Sex worker: 7; *P < 0.05

#### Contraceptive methods used at first sex and in current relationship

Half of ever sexually active young people who were not planning a pregnancy had used a modern method of contraception at the time of their first sexual intercourse and of these, the majority (48%) had used condoms (Table 2). Condoms were also the most commonly used method amongst currently sexually active youth, with 66% of participants using a modern contraceptive method: 39% were using condoms followed by 28% injectables and 8% the oral contraceptive pill.

**Table 2:** Percentage distributions of modern contraceptive use and method mix at first sexual relationship and current use among sexually active young people in the Kathmandu valley

Contraceptive method mix	n^a^	Percent	95% Confidence Interval
Used at first sexual intercourse			
Traditional method users	38	7.7	4.7–12.4
Non-users	206	41.9	35.9–48.0
Any modern method users	248	50.4	45.0–55.8
Condom	235	47.7	42.4–53.1
Pill^b^	12	2.5	1.4–4.4
Injectables	1	0.2	0–2.5
Total	492	100.0	-
Current use of contraception			
Traditional method users	40	20.8	16.0–26.5
Non-users	26	13.0	8.6–19.4
Any modern method users	129	66.2	59.7–72.1
Condom	77	39.4	32.1–47.2
Pill	10	4.9	2.6–8.8
Injectables	36	18.7	13.6–25.2
IUCD/Implants^c^	4	2.1	0.7–6.4
Sterilisation	2	1.1	0.3–3.4
Total	195	100.0	-

^a^Weighted samples to represent population; ^b^Emergency Contraceptive: 2; ^c^2 each

#### Determinants of contraceptive use

*Use at first sexual intercourse:* Seventy percent of males and 31% of females used modern contraceptive methods at their first sexual intercourse. The factors statistically associated with contraceptive use at first sexual intercourse were sex of participant, whether or not they had ever studied at school, age at first sexual intercourse and type of relationship (Table 3). The analysis suggests that compared to females, males had a 5.14 times greater odds of reporting use of contraception in their first sexual contact. Greater odds of contraceptive use were also associated with ever having studies at school, and having a relationship with a person other than a spouse. Those who had their first sexual encounter between the ages of 12–15 had significantly lower odds of having used contraception compared to women whose first sex occurred between 16 and 19 or 20 and 24 years. In the multivariable analysis only age at first sexual intercourse and relationship type with partner remained significant (Table 3).

**Table 3:** Factors associated with use of modern contraception at first sexual intercourse among sexually active Nepalese youth, univariate and multivariate analyses

Background Characteristics	n = 492 (%)	% of youth used modern contraceptive methods	*Univariate analyses*		*Multivariate analyses*	
			Odd ratio (95% CI)	*p*-value	Adjusted odd ratio (95% CI)	*p*-value
Sex						
Female	245 (49.8)	76 (30.9)	Ref.	<0.001*	Ref.	0.437
Male	247 (50.2)	172 (69.7)	5.14 (3.34–7.89)		1.48 (0.54–4.03)	
Place of origin						
Rural	296 (60.2)	141 (47.6)	Ref.		Ref.	0.849
Urban	196 (39.8)	107 (54.6)	1.32 (0.92–1.90)	0.128	0.96 (0.64–1.45)	
Ever studied at school						
Yes	469 (95.3)	245 (52.2)	6.51 (1.51–28.08)	0.013*	2.74 (0.74–10.21)	0.128
No	23 (4.7)	3 (14.4)	Ref.		Ref.	
Ethnicity						
Tibeto-Burmese	214 (43.6)	103 (41.6)	Ref.	0.363	Ref.	0.486
Indo-Aryan	278 (56.4)	145 (58.4)	1.18 (0.82–1.69)		1.17 (0.74–1.85)	
Religion						
Hindu	400 (81.3)	204 (82.1)	Ref.	0.671	Ref.	0.860
Non-Hindu^a^	92 (18.7)	44 (17.9)	0.91 (0.91–1.45)		0.95 (0.54–1.68)	
Age at the first sexual intercourse						
12–15	42 (8.5)	7 (2.6)	0.17 (0.07–0.41)	0.001*	0.10 (0.03–0.31)	0.001*
16–19	306 (62.3)	167 (67.5)	1.13 (0.72–1.76)		0.81 (0.50–1.33)	
20–24	144 (29.2)	74 (29.9)	Ref.		Ref.	
Relationship type with partner						
Spouse	255 (51.7)	76 (30.6)	Ref.	<0.001*	Ref.	0.003*
Boy/girlfriend	215 (43.8)	153 (61.7)	5.78 (3.88–8.61)		4.55 (1.63–12.75)	
Familiar person/sex worker^b^	22 (4.5)	19 (7.7)	15.29 (4.88–47.89)		11.85 (2.70–43.54)	
Type of participation						
Willingly	458 (93.0)	236 (95.1)	Ref.	0.064	Ref.	0.826
Unwillingly/forcefully	34 (7.0)	12 (4.9)	0.51 (0.25–1.04)		0.92 (0.44–1.95)	
Total	**-**	248 (50.4)				

^a^Buddhist: 69, Kirat: 12, Christian: 7, Islam: 4; ^b^Familiar person: 19, Sex worker: 7; CI: Confidence Interval; Ref: Reference category; *p < 0.05

*Current use:* Among young people not wanting to conceive, univariate analysis revealed greater odds of current use of contraception amongst males compared to females and amongst non-Hindus (Table 4). In the adjusted multivariate logistic we included all the variables with a *p*-value less than 0.25 in the univariate analysis that comprised sex, age, place of origin, religion, source of income and ever discussed about safer sex. The only significant association that remained after multivariable logistic regression analysis was with religion and current use of contraception, such that young people who belong to non-Hindu religious group had 3.24 (95% CI: 1.39-7.56; *p* = 0.008) times greater odds of using modern contraception compared to young people who stated they were Hindu.

**Table 4:** Results of univariate and multivariate analyses for current use of contraception among sexually active Nepalese youths

Background Characteristics	n = 195 (%)	% of youth currently using modern contraceptive	*Univariate analyses*		*Multivariate analyses*	
			Odd ratio/CI	*p*-value	Adjusted odd ratio (95% CI)	*p*-value
Sex:						
Female	151 (77.3)	94 (62.4)	Ref.	0.043*	Ref.	0.232
Male	44 (22.7)	35 (79.0)	2.27 (1.03–5.03)		1.62 (0.73–3.61)	
Age (in years):						
15–19	29 (14.7)	16 (55.3)	0.58 (0.25–1.34)	0.195	0.63 (0.26–1.51)	0.287
20–24	166 (85.3)	113 (68.0)	Ref.		Ref.	
Marital Status:						
Ever married	160 (82.1)	104 (65.2)	Ref.	0.487	-	-
Never married	35 (17.9)	25 (70.7)	1.29 (0.62–2.68)			
Place of origin:						
Rural	122 (62.8)	76 (62.1)	Ref.	0.154	Ref.	0.087
Urban	73 (37.2)	53 (73.0)	1.64 (0.82–3.29)		1.87 (0.91–3.83)	
Education:						
Some secondary and below^a^	82 (41.9)	54 (65.7)	Ref.	0.927	-	-
SLC and above	113 (58.1)	75 (66.4)	1.03 (0.52–2.05)			
Ethnicity:						
Tibeto-Burmese	90 (45.9)	59 (66.0)	Ref.	0.963	-	-
Indo-Aryan	105 (54.1)	70 (66.3)	1.01 (0.60–1.69)			
Religion:						
Hindu	159 (81.6)	99 (61.9)	Ref.	0.007*	Ref.	0.008*
Non-Hindu^b^	36 (18.4)	30 (85.0)	3.49 (1.43–8.49)		3.24 (1.39–7.56)	
Occupation:						
Non-students	139 (71.4)	93 (66.9)	Ref.	0.663		
Students	56 (28.6)	36 (64.3)	0.89 (0.53–1.50)			
Source of income:						
Parents and others	179 (92.1)	116 (64.6)	Ref.	0.081	Ref.	0.209
Self	16 (7.9)	13 (84.5)	2.99 (0.97–10.34)		2.18 (0.63–7.53)	
Age at the first sexual intercourse						
12–15	19 (9.4)	11 (60.3)	0.61 (0.18–2.02)	0.582	-	-
16–19	106 (54.5)	68 (63.7)	0.70 (0.34–1.43)			
20–24	70 (36.1)	50 (71.4)	Ref.			
Relationship type						
Spouse	158 (81.3)	103 (64.8)	Ref.		-	-
Boy/girlfriend or causal partner^c^	37 (18.7)	26 (71.9)	1.39 (0.69–2.81)	0.348		
No. of sexual partners in life time						
One	170 (87.5)	113 (66.4)	Ref.	0.871	-	-
Two and more	24 (12.5)	16 (64.7)	0.93 (0.37–2.32)			
Ever discussed of safe sex with partner						
No	37 (19.1)	21 (55.6)	Ref.	0.111	Ref.	0.444
Yes	158 (80.9)	108 (68.7)	1.75 (0.87–3.50)		1.34 (0.62–2.88)	
Ever discussed of contraceptive use						
No	21 (10.6)	14 (69.1)	Ref.	0.720	-	-
Yes	174 (89.4)	115 (65.8)	0.86 (0.37–2.01)			
Source of SRH information						
Radio	148 (76.1)	100 (67.1)	Ref.	0.858	-	-
Television	35 (17.8)	22 (63.7)	0.86 (0.36–2.06)			
Friends, teachers and others	12 (6.1)	7 (61.7)	0.79 (0.29–2.14)			
Total	**-**	129 (66.2)				

^a^No education: 5; Primary 22; ^b^Buddhist: 28; Christian: 5; Kirat: 2; Islam: 1; ^c^Boy/girlfriend: 34, Causal partner: 3; Ref: Reference category; *P < 0.05

## Discussion

This study examined the timing, circumstances and use of modern contraception among sexually active youth aged 15–24 living in urban area of the Kathmandu Valley at their first and in their current sexual relationship. We found that half of young people had used a modern method of contraception at first intercourse and over two thirds in their current relationships and in both circumstances condoms were the most frequently used method followed by the oral contraceptive pill and injectables. These findings are similar to those in the 2011 National Adolescents and Youth Survey figures that revealed 46% of young people had used contraception at the time of their first sexual intercourse and of those, 91% of them used condoms, three precent the oral contraceptive pill, and one percent the injection [15]. This may relate to the fact that government of Nepal has made these methods easily available and accessible to all reproductive age men and women in Nepal. As a policy measure, findings of this study are very useful as it can inform policy and decision making in the government healthcare system to increase the contraception users among youth to protect against unintended pregnancy and sexually transmitted infections.

The use of one of the modern contraceptive methods at first sexual encounter was, in univariate analysis, significantly associated with young people’s sex, schooling, age at first sexual encounter and relationship type. However, in the multivariate analysis only age at first sexual intercourse and relationship type with a partner was statistically significantly associated with modern contraceptive use. Although it was expected that socio-cultural factors such as religion and ethnicity might influence the use of modern contraceptive use among youth, however, it did not have any significant association with use of contraception at first sexual relationship.

In contrast use of a modern method of contraception was associated with religion with significantly less odds of using a modern method of contraception if the young person identified as Hindu. Other studies have similarly reported that socio-cultural factors such as religion influence sexual attitudes and behaviour including utilisation of contraception [13, 29–32]. The possible reason for greater use of contraception by non-Hindu youths, primarily Buddhist and Christian, may relate to the more egalitarian nature of their religious and cultural beliefs and practices compared to the Hindu mores [32]. Studies have shown that there is a strong preference for childbearing immediately after marriage in Hindu societies to demonstrate that the partnership is a good one and women may not be educated about contraceptive options until after the birth of the first child [33, 34].

Although not demonstrated in our study, other research in Nepal and elsewhere has consistently shown that young people with higher education are more likely to use contraception than those who have little or no education [13, 17]. Reasons for our finding are possibly due to the sample, who were an urban population and who were likely to be aware of contraception and its benefits though exposure to mass media. This sample also excluded young women wishing to conceive who were likely to be less educated as they had chosen marriage and childbirth over tertiary studies.

Several limitations of the study should be noted. Young people provided information about their sexual relationship and contraceptive use retrospectively and there is the possibility that recall bias, social desirability and underreporting affected their responses. Another limitation of this study concerns the sample of participants. This study is limited to sexually active young people living in urban areas of the Kathmandu valley, and therefore, the generalizability of this study’s findings is restricted. The responses to the question on use of contraceptives are self-reported and the validity of the respondents’ claims has not been ascertained. However, the study team made a concerted effort to improve the reliability of the data by undertaking the interviews in total privacy, and using validated questions.

## Conclusions

This study shows that majority of youth at their first and current sexual contact used condom to protect against unintended pregnancy and sexually transmitted infections. Age at first sexual intercourse and relationship type with a partner were associated with use of modern contraceptive first sex, whereas religion was found to be associated with current modern contraceptive use. Our results present evidence that may be useful to inform future policy direction about how to increase young people’s access to contraception in Nepal. In particular sex education needs to begin early as it was the youth initiating sexual intercourse under the age of 16 who were least likely to use a modern method of contraception. Young people need better skills to be able to negotiate sexual and contraceptive decisions in their relationship and the problems of high prevalence of child marriage and early childbearing that needs to be addressed if Nepal is to reach the millennium development goal in terms of gender inequality and empowerment of women.

## Abbreviations

CI: Confidence interval
HIV: Human immuno-deficiency virus
SPSS: Statistical package for the social sciences
USA: United States of America.
